# Nanoparticles of novel organotin(IV) complexes bearing phosphoric triamide ligands

**DOI:** 10.3762/bjnano.4.11

**Published:** 2013-02-12

**Authors:** Zahra Shariatinia, Ebadullah Asadi, Vahid Tavasolinasab, Khodayar Gholivand

**Affiliations:** 1 Department of Chemistry, Amirkabir University of Technology (Polytechnic), P. O. Box 159163-4311, Tehran, Iran; 2 Department of Chemistry, Tarbiat Modarres University, P. O. Box: 14115-175, Tehran, Iran

**Keywords:** luminescence, nanoparticles, organotin(IV) complexes, phosphoric triamide, ultrasonic

## Abstract

Four novel organotin(IV) complexes containing phosphoric triamide ligands were synthesized and characterized by multinuclear (^1^H, ^31^P, ^13^C) NMR, infrared, ultraviolet and fluorescence spectroscopy as well as elemental analysis. The ^1^H NMR spectra of complexes **1**–**4** proved that the Sn atoms adopt octahedral configurations. The nanoparticles of the complexes were also prepared by ultrasonication, and their SEM micrographs indicated identical spherical morphologies with particles sizes about 20–25 nm. The fluorescence spectra exhibited blue shifts for the maximum wavelength of emission upon complexation.

## Introduction

In recent years, the increasing progress in the preparation of nanomaterials has led to characterization of a great number of nanostructures [1]. Nanoscale materials are of significance owing to their small sizes and large specific surface areas indicating novel properties that differ considerably from those of the corresponding bulk materials [2–3]. The coordination chemistry of organotin(IV) complexes has become of great interest due to the wide applications of these coordination compounds [4–6]. For example, they can act as potential antitumor agents [7–8], wood preservatives, agrochemical fungicides and biocides [9–10], as well as catalysts [11]. The organotin(IV) complexes with phosphorus-based ligands bearing the P(E) group (E = O, S, or Se) are especially important because of their various coordination numbers [12–14]. The central tin atom presents a diverse coordination environment depending on the different nature of substituents [15]. Hypervalent octahedral geometries were observed for the Sn(IV) atoms in organotin(IV) complexes of O-donor ligands, such as phosphoramidates [16–22], imidodiphosphonic acids [23] and bis(diphenylphosphino) pyridine [24], while a trigonal bipyramidal coordination was found for Sn atoms in thiophosphinates complexes [25].

Ultrasonic vibration has the potential to be a simple and effective process to produce homogenous nanomaterials. Some investigations have proved that ultrasonication can be applied to prepare nanosized coordination compounds [26–29]. The synthesis of nanoplates of a cadmium(II) coordination polymer by a sonochemical process was reported [30]. It is notable that, as far as we know, there are no reports about the preparation of nanosized organotin(IV) complexes bearing phosphoric triamide ligands.

In this work, following on from our previous works to prepare organometallic compounds including phosphoric triamide ligands [17–18], novel organotin(IV) complexes with the formula SnCl_2_(CH_3_)_2_L^1^_2_ (**1**), SnCl_2_(OH_2_)_2_L^1^_2_ (**2**), SnCl(C_6_H_5_)_3_L^1^_2_ (**3**) and SnCl_2_(CH_3_)_2_L^2^_2_ (**4**), where L^1^ = C_6_H_5_C(O)NHP(O)[NC_4_H_8_N(C_6_H_5_)]_2_ (**5**) and L^2^ = P(O)[NC_4_H_8_N(C_6_H_5_)]_3_ (**6**), were synthesized and characterized. The results of NMR, IR, UV and fluorescence spectroscopy of complexes **1**–**4** were compared with each other and their related phosphoric triamide ligands **5**, **6**. The spherical nanoparticles of complexes **1**–**4** were obtained by ultrasonication with particle sizes of about 20–25 nm.

## Results and Discussion

### Spectroscopic study

**Scheme 1:**
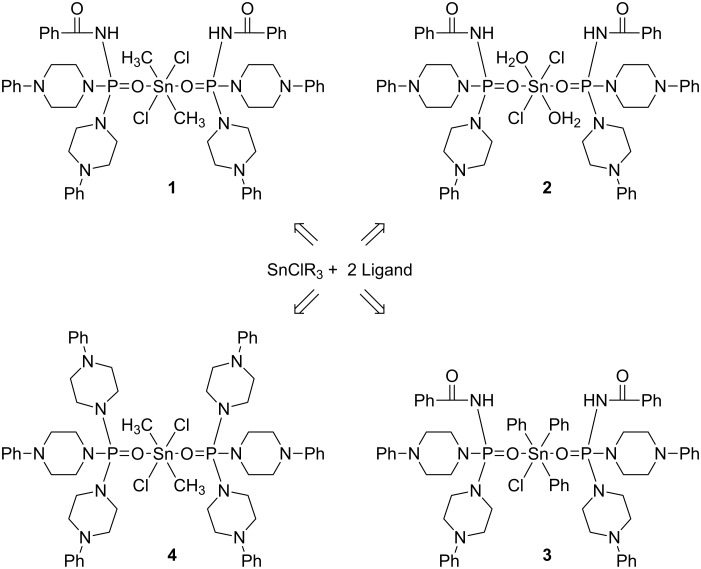
The preparation pathway of organotin(IV) complexes **1**–**4**.

In this work, new organotin(IV) complexes **1**–**4** were synthesized from the reaction of SnClR_3_ with phosphoric triamide ligands (Scheme 1). A summary of the NMR and IR parameters of complexes **1**–**4** and their corresponding ligands C_6_H_5_C(O)NHP(O)[NC_4_H_8_N(C_6_H_5_)]_2_ (**5**) [31], P(O)[NC_4_H_8_N(C_6_H_5_)]_3_ (**6**) [32] and also of similar organotin(IV) complexes **7**–**13** reported earlier [14–15,17–18] are given in Table 1.

**Table 1:** Selected spectroscopic NMR and IR data of compounds **1**–**13**.

compound^a^	δ(^31^P) (ppm)	δ(^119^Sn) (ppm)	^2^*J*(^119^Sn,H)/^2^*J*(^117^Sn,H) (Hz)	ν(P=O) (cm^−1^)	ν(C=O) (cm^−1^)	Ref.

ML^1^_2_ (**1**)	9.33	−166.77	55.1	1137	1671	—^d^
SnCl_2_(OH_2_)_2_L^1^_2_ (**2**)	11.21	—	—	1137	1675	—^d^
SnCl(C_6_H_5_)_3_L^1^_2_ (**3**)	11.32	—	—	1190	1667	—^d^
ML^2^_2_ (**4**)	17.33	—	83.4	1135	—	—^d^
C_6_H_5_C(O)NHP(O)[NC_4_H_8_N(C_6_H_5_)]_2_ (**5**)	9.37	—	—	1205	1675	[31]
P(O)[NC_4_H_8_N(C_6_H_5_)]_3_ (**6**)	18.50	—	—	1189	—	[32]
M[4-F-C_6_H_4_C(O)NHP(O)(NC_5_H_10_)_2_]_2_ (**7**)	10.15^b^	−210.75	111.4/42.1^b^	1162	1680	[17]
M[C_6_H_5_C(O)NHP(O)(NC_4_H_8_)_2_]_2_ (**8**)	6.85^b^	—	111.8/43.7^b^	1115	1672	[18]
M[C_6_H_5_C(O)NHP(O)(NH-C(CH_3_)_3_]_2_ (**9**)	2.95^b^	—	110.9/42.1^b^	1150	1648	[18]
M[3-N-C_6_H_4_C(O)NHP(O)(NH-C(CH_3_)_3_)_2_]_2_ (**10**)	2.45^b^	−166.77	114.0/110.8^b^	1225	1683	[20]
M[3-N-C_6_H_4_C(O)NHP(O)(NHC_6_H_11_)_2_]_2_ (**11**)	5.68^b^	−166.77	71.2/68.2^b^	1202	1649	[20]
M[4-N-C_6_H_4_C(O)NHP(O)(NHC_6_H_11_)_2_]_2_ (**12**)	5.48^b^	−238.25	—	1163	1676	[20]
M[C_6_H_5_P(O)(NHCH(CH_3_)_2_]_2_ (**13**)	18.38^c^	—	88.9/–	1138	—	[21]

^a^M = SnCl_2_(CH_3_)_2_, L^1^ = compound **5**, L^2^ = compound **6**. ^b^(DMSO), ^c^(CHCl_3_), ^d^This work.

Comparing the phosphorus chemical shift, δ(^31^P), of compounds **1**–**4** demonstrates that it is the most deshielded atom in **4** (containing three *N*-phenylpiperazinyl substituents on the P atom with δ(^31^P) = 17.33 ppm). The ^31^P NMR of complexes **1**–**3**, each containing identical phosphoric triamide ligands, show that the phosphorus atom is at the most upfield region in **1** with M = SnCl_2_(CH_3_)_2_. Further, the δ(^31^P) shifts downfield from **1** to **3**. The complexes **1** and **4** both contain SnCl_2_Me_2_, and they differ in the phosphoric triamide ligands. The ^31^P NMR reveals that the phosphorus atom in **1** appears at a much more upfield region than that of **4**, which is due to the presence of more electron-donating phosphoric triamide ligands in **1**. The δ(^31^P) in complex **1** and its corresponding ligand **5** are observed at about 9.00 ppm, while those of complex **4** and its ligand **6** are at about 18.00 ppm. These downfield shifts in compounds **4** and **6** show that the *N*-benzoyl substituents cause more electron donation to the phosphorus atoms than do the 4-phenylpiperazinyl moieties.

**Figure 1:**
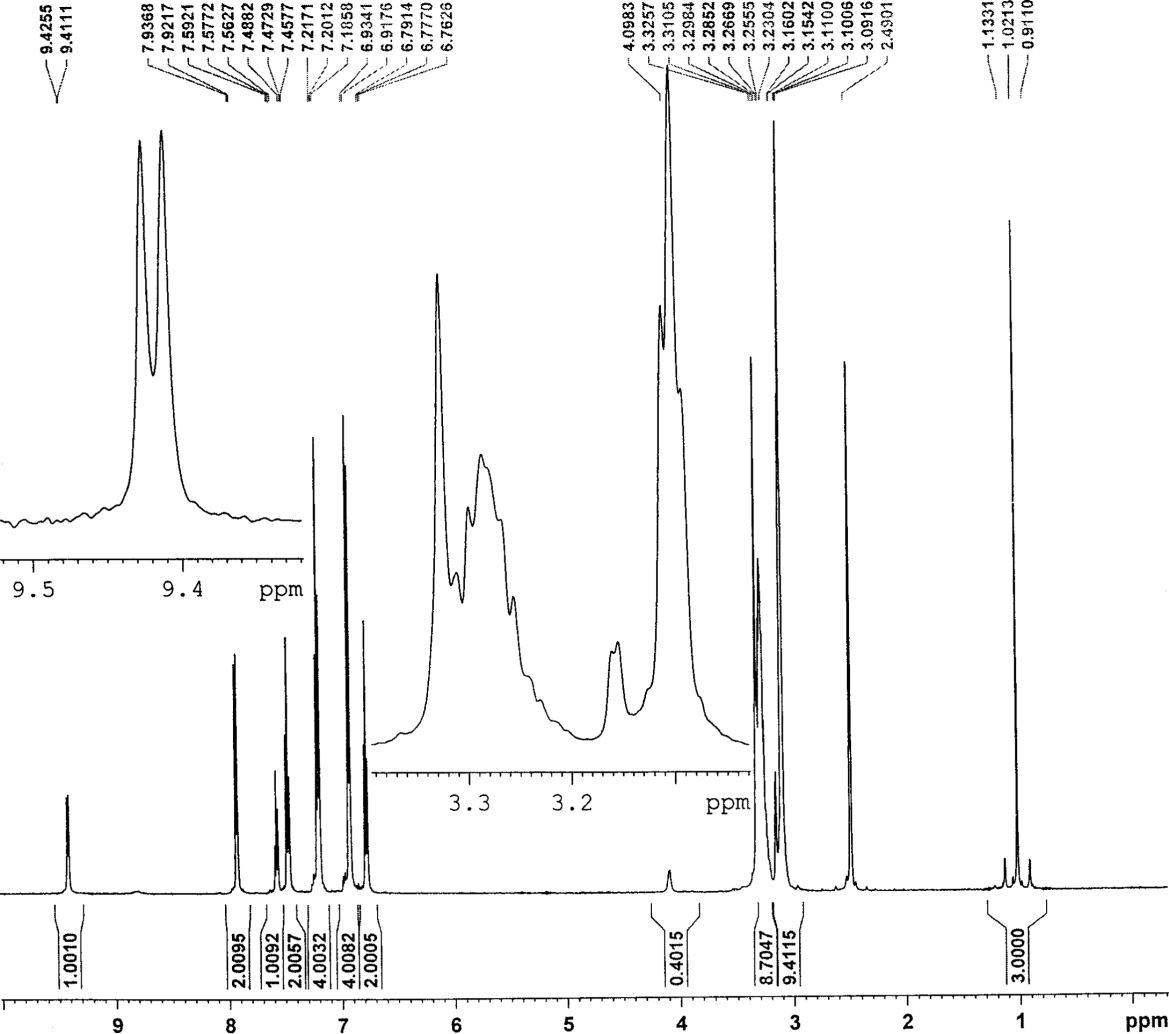
The ^1^H NMR spectrum of compound **1**.

**Figure 2:**
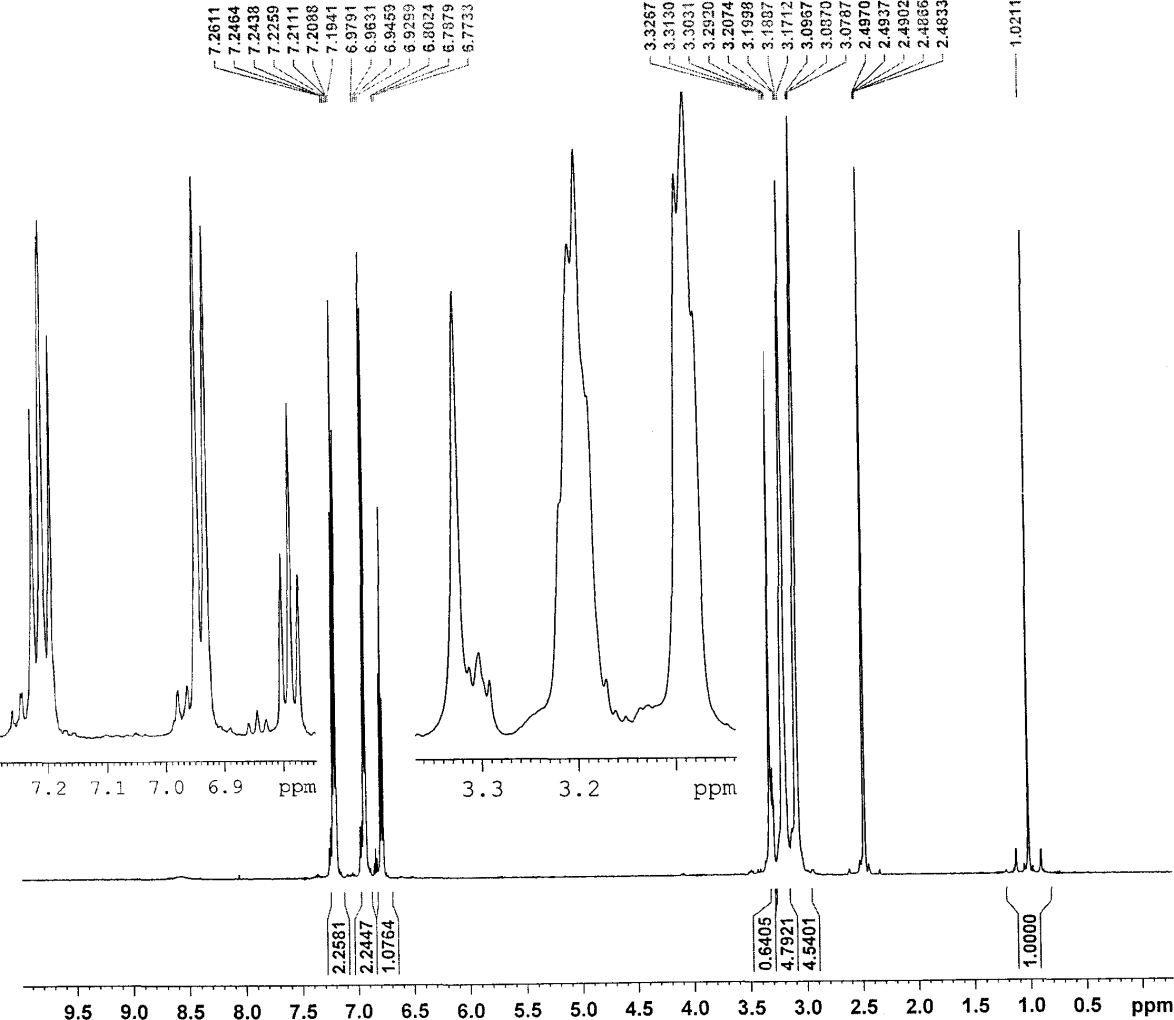
The ^1^H NMR spectrum of compound **4**.

It can be deduced from the ^1^H NMR spectra of complexes **1**–**4** that the Sn atoms adopt octahedral configurations (Figure 1 and Figure 2). The coordination number of the central Sn atom with different phosphoric triamide ligands can change. For example, Jurkschat et al. prepared [33–35] several organotin(IV) complexes of HMPA, P(O)(NMe_2_)_3_, in which the Sn atoms indicate distorted trigonal bipyramidal geometries. The ^1^H NMR spectra of compounds **1** and **4** exhibit the ^2^*J*(^119^Sn,H) = 55.1, 83.4 Hz for the geminal coupling of the ^119^Sn atom with the hydrogen atoms of the CH_3_ groups. The ^2^*J*(^119/117^Sn,H) coupling constants were obtained in the range of 114.0/110.8 Hz (for **10**) to 71.2/68.2 Hz (for **11**), see Table 1.

The IR spectra indicate that the ν(P=O) of complexes **1**–**4** are weaker than their corresponding ligands **5** and **6** due to the formation of Sn–O–P bonds. Also, the ν(C=O) of complexes **1–3** are nearly equal to (or slightly weaker than) the ν(C=O) value in ligand **5** (1675 cm^−1^). The bands at about 560 and 520 cm^−1^ correspond to asymmetric and symmetric stretching frequencies of Sn–C bonds. Also, the bands at ≈450 cm^−1^ are for the stretching frequencies of Sn–O bonds.

### SEM and fluorescence studies

**Figure 3:**
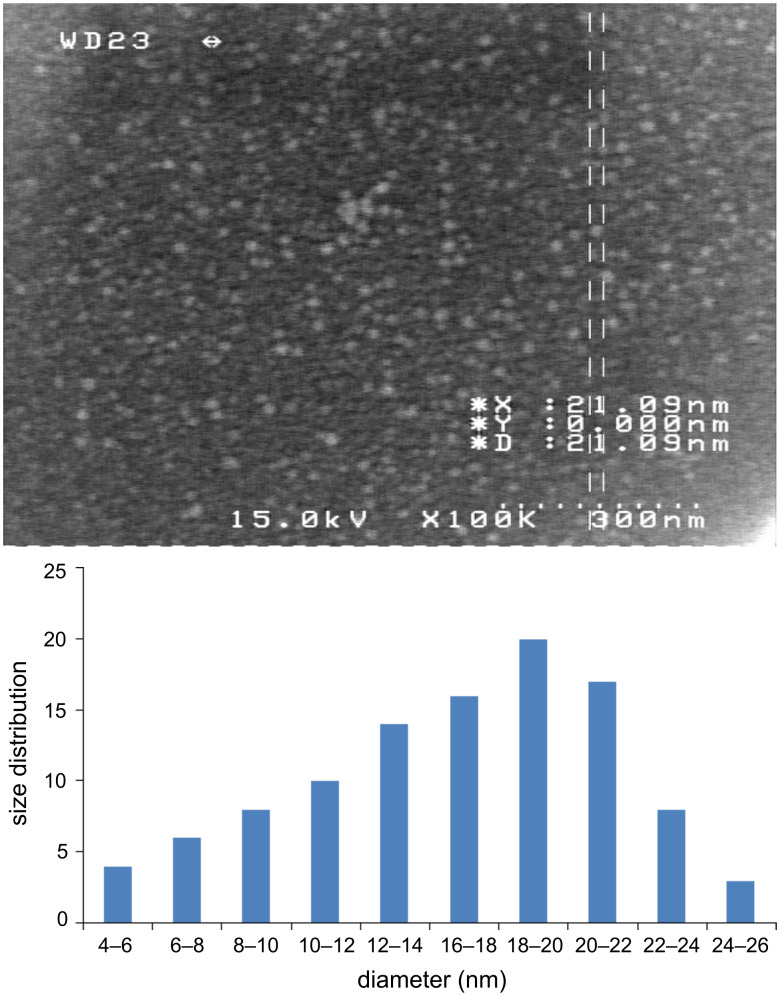
SEM micrograph of compound **1** and histogram indicating the size distribution in the SEM images.

**Figure 4:**
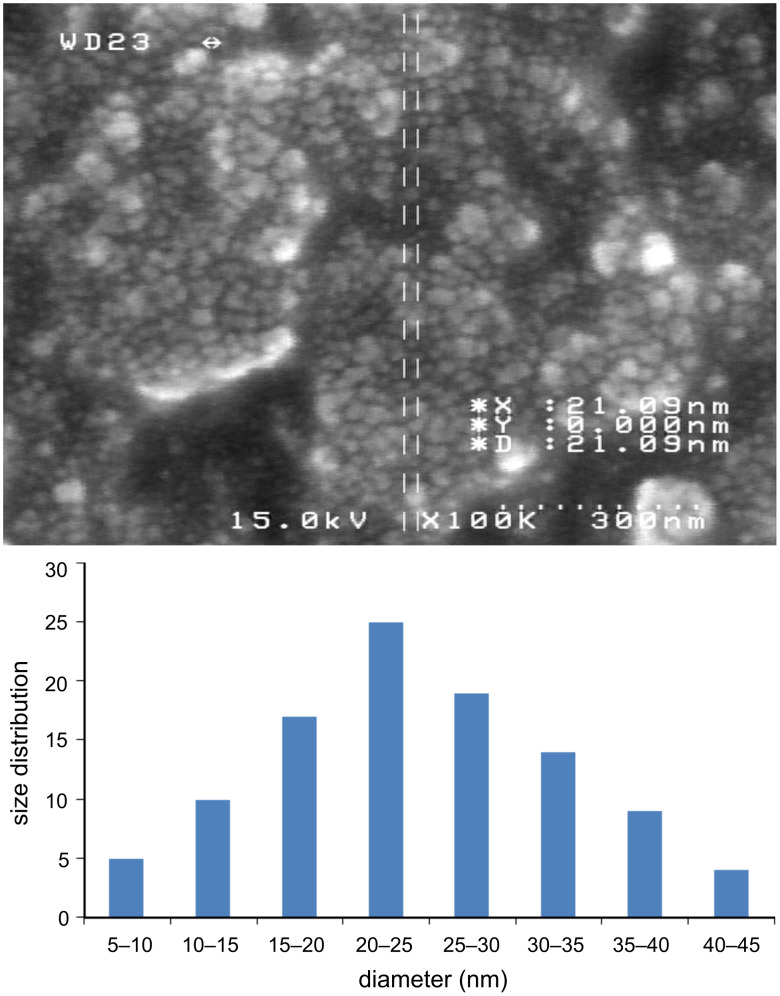
SEM micrograph of compound **2** and histogram indicating the size distribution in the SEM images.

**Figure 5:**
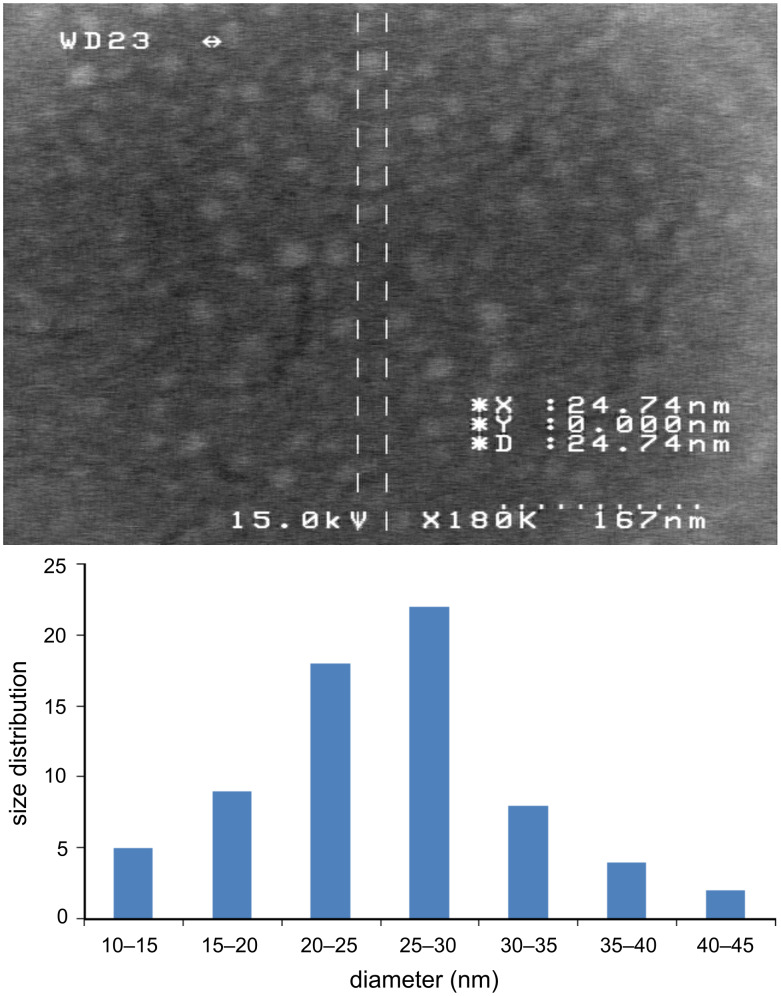
SEM micrograph of compound **3** and histogram indicating the size distribution in the SEM images.

**Figure 6:**
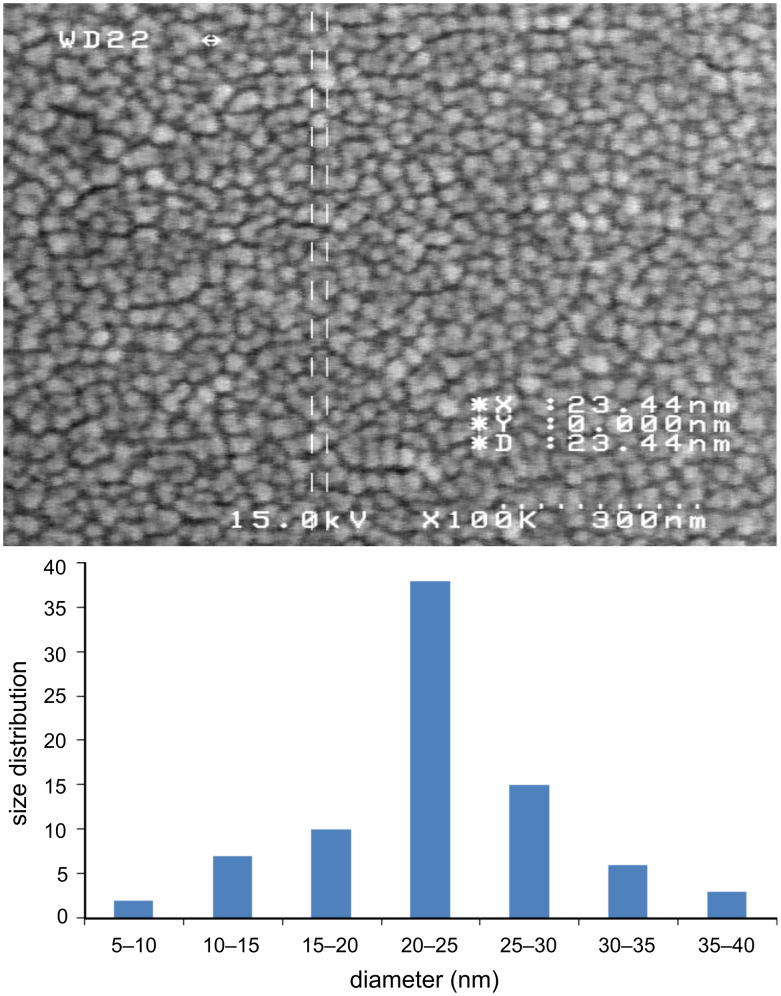
SEM micrograph of compound **4** and histogram indicating the size distribution in the SEM images.

**Figure 7:**
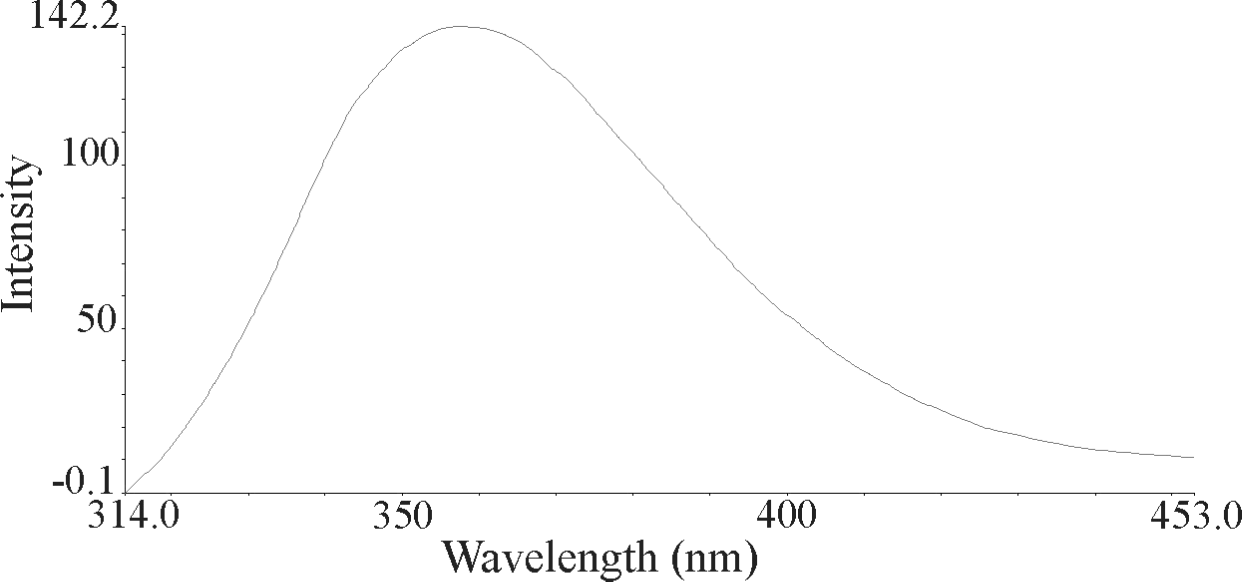
Fluorescence spectrum of compound **1** in methanol.

**Figure 8:**
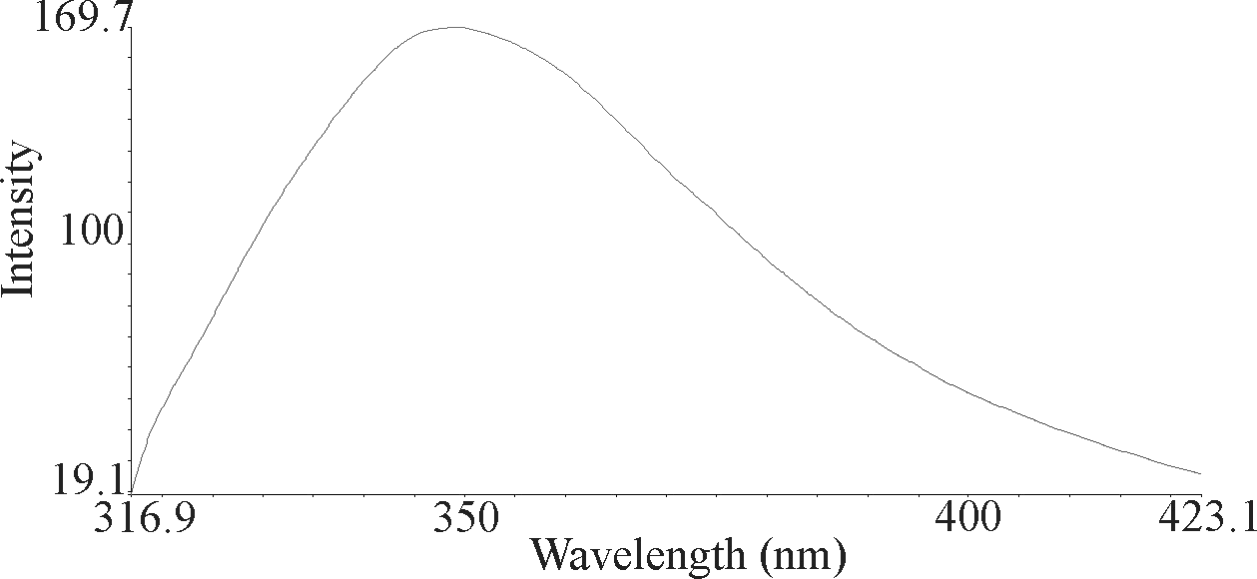
Fluorescence spectrum of compound **2** in methanol.

**Figure 9:**
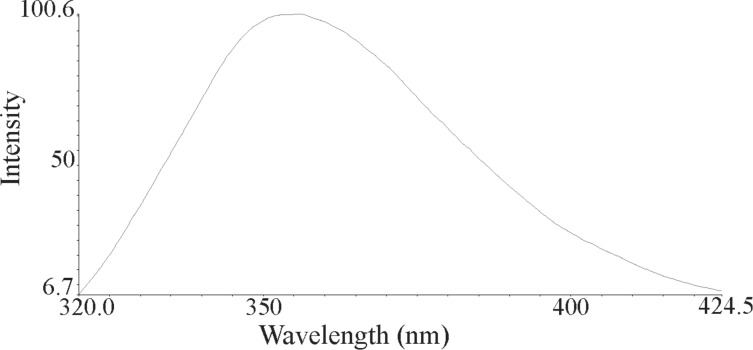
Fluorescence spectrum of compound **3** in methanol.

**Figure 10:**
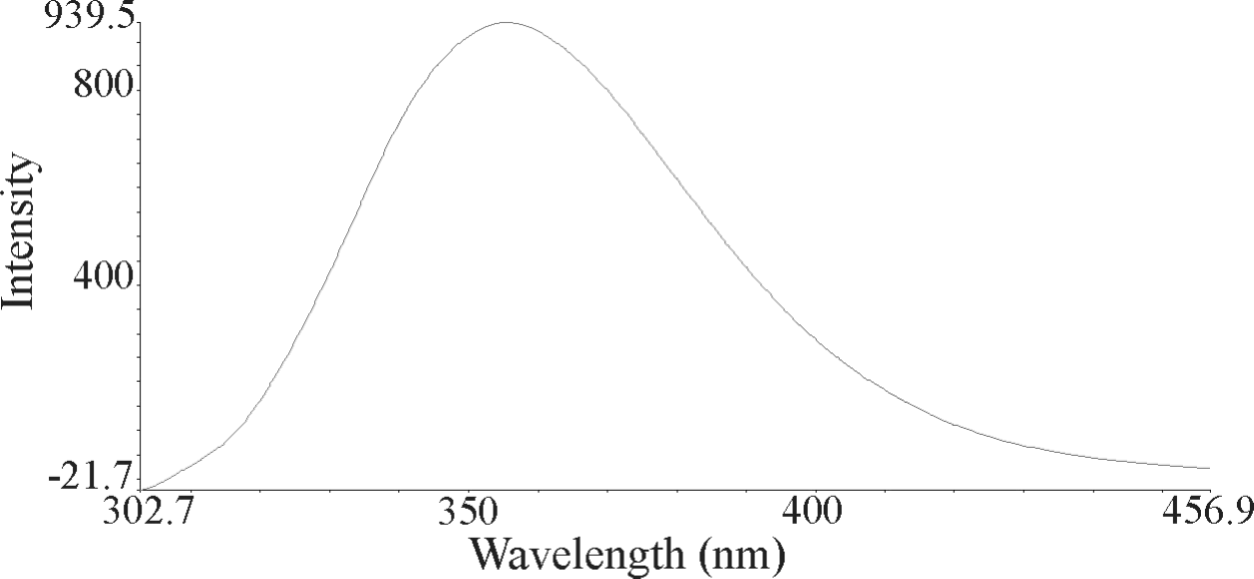
Fluorescence spectrum of compound **4** in methanol.

**Figure 11:**
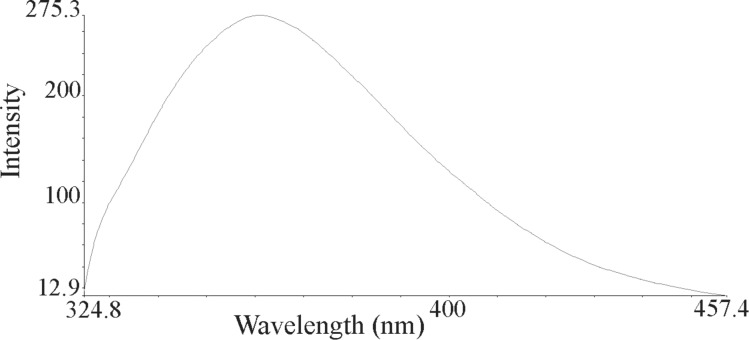
Fluorescence spectrum of compound **5** in methanol.

**Figure 12:**
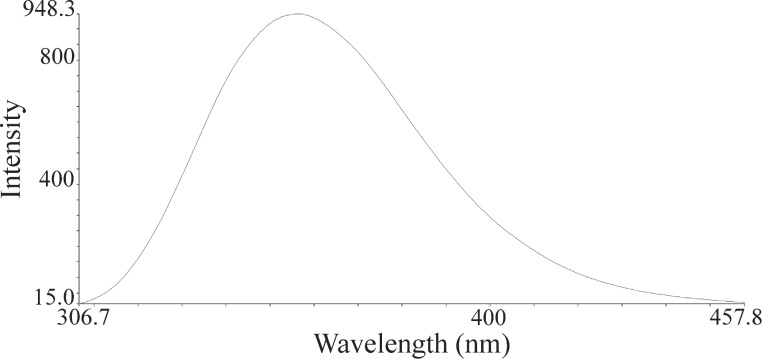
Fluorescence spectrum of compound **6** in methanol.

The nanoparticles of complexes **1**–**4** were prepared by ultrasonication. In this way, the synthesis of these compounds was performed in an ultrasonic bath at 30 °C for about 1–2 h. The SEM micrographs of the nanoparticles are shown in Figures 3–6 indicating that the particle sizes are about 20–25 nm with identical spherical morphologies of the nanoparticles. It is notable that SEM micrographs could be obtained from both dissolved and powdered samples. For the dissolved samples, a few droplets were placed on a small piece of foil, whereas the powdered compounds were directly placed on the sample holder. Here, the complexes were dissolved in methanol, and after evaporation of the solvent, the SEM images were obtained from the nanoparticles prepared on aluminum foil. The fluorescence spectra of complexes **1**–**4** and their related phosphoric triamide ligands **5** and **6** are represented in Figures 7–12 (see below), respectively. Also, a summary of the UV–vis and fluorescence spectra of compounds **1**–**6** is given in Table 2. The results reveal that the UV absorption wavelengths in these compounds vary from 280 to 330 nm, which are related to the interligand π→π* and n→π* electronic transitions.

**Table 2:** The summary of fluorescence and UV spectra of compounds **1**–**6**.

compound	λ(max) of excitation (nm)	λ(max) of emission (nm)	Maximum intensity of emission	λ(max) of absorption in UV (nm)

**1**	295.0	355.5	142.2	291–296
**2**	300.0	352.0	169.7	282–310
**3**	300.0	353.5	100.6	286–330
**4**	295.0	358.5	939.5	282–300
**5**	310.0	360.0	275.3	282–297
**6**	285.0	360.0	948.3	287–313

The fluorescence spectra of each compound were repeated at various wavelengths from about 220 to 360 nm to find the maximum emission intensity. The emission signals with maximum intensities are shown in Figures 7–12. It can be seen that the λ(max) of emission appears at 360 nm for both ligands **5** and **6** while it decreases in their corresponding complexes (blue shift). This observation shows that the band gap (Δ*E*) increases upon complexation. Moreover, the peak intensities are very much smaller in complexes **1**–**3** relative to that of their corresponding ligand **5**. However, the λ(max) of emission and the peak intensity are very close to each other in compound **4** and its phosphoric triamide ligand **6**.

## Experimental

### Spectroscopic measurements

The ^1^H, ^13^C and ^31^P spectra were recorded on a Bruker Avance DRS 500 spectrometer. ^1^H and ^13^C chemical shifts were determined relative to internal TMS and ^31^P chemical shifts relative to 85% H_3_PO_4_ as an external standard. Infrared (IR) spectra were recorded on a Shimadzu model IR-60 spectrometer. Elemental analysis was performed by using a Heraeus CHN-O-RAPID apparatus. Melting points were obtained with an Electrothermal instrument. The scanning electron microscopy (SEM) micrographs were obtained with a Philips instrument (XL30), under vacuum, accelerated at 30 kV. The fluorescence and UV–visible spectra were measured by using a Perkin Elmer LS55 instrument and a Genway spectrophotometer, respectively.

### Synthesis

#### Bis[*N*-benzoyl-bis(4-phenylpiperazin-1-yl)phosphinic amide-κ*O*]dichlorido dimethyltin(IV) (**1**)

A solution of dimethyltin(IV) dichloride (0.5 mmol, 0.110 g) in dry methanol (20 mL) was added dropwise to a solution of *N*-benzoyl-bis(4-phenylpiperazin-1-yl)phosphinic amide (1 mmol, 0.489 g) at room temperature. After two hours stirring, the solution flask was placed in an ultrasonic bath at 30 °C for two hours. Then, the solution was evaporated and the nanoparticles of the product were dried. The product was further purified by washing with diethylether and CCl_4_. mp 122–124 °C; yield: 55%; anal. calcd for C_56_H_70_Cl_2_N_10_O_4_P_2_Sn: C, 56.11; H, 5.88; N, 11.68; found: C, 56.08; H, 5.87; N, 11.69; ^119^Sn{^1^H} NMR (DMSO-*d*_6_): δ −166.77 (s); ^31^P{^1^H} NMR (DMSO-*d*_6_): δ 9.33 (s); ^1^H NMR (DMSO-*d*_6_): δ 0.91 (d, ^2^*J*(^119^Sn, H) = 55.1 Hz, Sn-CH_3_), 1.02 (s, 6H, Sn-CH_3_), 3.11 (m, 16H, CH_2_), 3.29 (m, 16H, CH_2_), 6.78 (t, ^3^*J*(H,H) = 8.3 Hz, 4H, Ar-H, phenylpiperazinyl), 6.92 (d, ^3^*J*(H,H) = 8.3 Hz, 8H, Ar-H, phenylpiperazinyl), 7.20 (t, ^3^*J*(H,H) = 8.3 Hz, 8H, Ar-H, phenylpiperazinyl), 7.47 (t, ^3^*J*(H,H) = 7.6 Hz, 4H, Ar-H, N-benzoyl), 7.58 (t, ^3^*J*(H,H) = 7.6 Hz, 2H, Ar-H, N-benzoyl), 7.92 (d, ^3^*J*(H,H) = 7.6 Hz, 4H, Ar-H, N-benzoyl), 9.41 (d, ^2^*J*(PNH) = 7.2 Hz, 2H, NH); ^13^C NMR (DMSO*-d*_6_): δ 22.36 (s, Sn-CH_3_), 40.10 (s), 48.85 (d, ^2^*J*(P,C) = 5.3 Hz, CH_2_), 115.66 (s), 119.07 (s), 128.19 (s), 128.27 (s), 128.88 (s), 132.18 (s), 133.60 (d, ^3^*J*(P,C) = 7.9 Hz, ipso-C), 151.14 (s), 168.37 (s, C=O); FTIR (KBr): 3437 (NH), 3071 (CH), 2906 (CH), 2840 (CH), 1671 (C=O), 1595, 1441, 1210, 1137 (P=O), 972 (P-N_amine_), 742 (P-N_amide_), 708, 532 (Sn-C)_s_ cm^−1^.

#### Bis[*N*-benzoyl-bis(4-phenylpiperazin-1-yl)phosphinic amide-κ*O*]diaqua dimethyltin(IV) (**2**)

To a solution of *N*-benzoyl-bis(4-phenylpiperazin-1-yl)phosphinic amide (1 mmol, 0.489 g) in methanol (15 mL), dichlorotin(IV) dihydrate (0.5 mmol, 0.113 g) was added at room temperature, and the mixture was stirred for one hour. Then the solution flask was placed in an ultrasonic bath at 30 °C for two hours. After evaporation of the solvent, the nanoparticles of the product were obtained. The powder product was further purified by washing with diethylether and CCl_4_. mp 143–145 °C; yield: 58%; anal. calcd for C_54_H_68_Cl_2_N_10_O_6_P_2_Sn: C, 53.84; H, 5.69; N, 11.63; found: C, 53.85; H, 5.70; N, 11.62; ^31^P{^1^H} NMR (CD_3_OD): δ 11.21 (s); ^1^H NMR (CD_3_OD): δ 3.29 (m, 16H, CH_2_), 3.48 (m, 16H, CH_2_), 6.92 (t, ^3^*J*(H,H) = 8.0 Hz, 4H, Ar-H, phenylpiperazinyl), 7.05 (d, ^3^*J*(H,H) = 8.0 Hz, 8H, Ar-H, phenylpiperazinyl), 7.27 (t, ^3^*J*(H,H) = 8.0 Hz, 8H, Ar-H, phenylpiperazinyl), 7.50 (t, ^3^*J*(H,H) = 7.5 Hz, 4H, Ar-H, *N*-benzoyl), 7.59 (t, ^3^*J*(H,H) = 7.5 Hz, 2H, Ar-H, N-benzoyl), 7.91 (d, ^3^*J*(H,H) = 7.5 Hz, 4H, Ar-H, *N*-benzoyl); ^13^C NMR (CD_3_OD): δ 45.80 (s, CH_2_), 51.97(s, CH_2_), 118.40 (s), 122.58 (s), 129.23 (s), 129.74 (s), 130.29 (s), 133.93 (s), 134.74 (d, ^3^*J*(P,C) = 8.9 Hz, ipso-C), 152.09 (s), 171.49 (s, C=O); FT-IR (KBr): 3424 (NH), 3062 (CH), 2833 (CH), 1675 (C=O), 1593, 1491, 1447, 1230, 1137 (P=O), 973 (P-N_amine_), 753 (P-N_amide_), 690, 465 (Sn-O) cm^−1^.

#### Bis[*N*-benzoyl-bis(4-phenylpiperazin-1-yl)phosphinic amide-κ*O*]chlorido triphenyltin(IV) (**3**)

*N*-benzoyl-bis(4-phenylpiperazin-1-yl)phosphinic amide (1 mmol, 0.489 g) was dissolved in methanol (15 mL), and triphenyltin(IV) chloride (0.5 mmol, 0.193 g) was added at room temperature and the mixture stirred for half an hour. Then the solution flask was placed in an ultrasonic bath at 30 °C for one hour. The evaporation of the solvent yielded the nanoparticles of the product, which was further purified by washing with diethylether and CCl_4_. mp 114–115 °C; yield: 54%; anal. calcd. for C_72_H_79_ClN_10_O_4_P_2_Sn: C, 63.37; H, 5.84; N, 10.26; found: C, 63.35; H, 5.83; N, 10.25; ^31^P{^1^H} NMR (CD_3_OD): δ 11.32 (s); ^1^H NMR (CD_3_OD): δ 3.32 (m, 16H, CH_2_), 3.43 (m, 16H, CH_2_), 6.84 (t, ^3^*J*(H,H) = 8.1 Hz, 4H, Ar-H, phenylpiperazinyl), 6.96 (d, ^3^*J*(H,H) = 8.1 Hz, 8H, Ar-H, phenylpiperazinyl), 7.23 (t, ^3^*J*(H,H) = 8.1 Hz, 8H, Ar-H, phenylpiperazinyl), 7.44 (d, ^3^*J*(H,H) = 5.9 Hz, 6H, Ar-H, Sn-Ph), 7.49 (t, ^3^*J*(H,H) = 5.9 Hz, 3H, Ar-H, Sn-Ph), 7.60 (t, ^3^*J*(H,H) = 5.9 Hz, 6H, Ar-H, Sn-Ph), 7.83 (t, ^3^*J*(H,H) = 7.0 Hz, 2H, Ar-H, *N*-benzoyl), 7.70 (t, ^3^*J*(H,H) = 7.0 Hz, 4H, Ar-H, *N*-benzoyl), 7.86 (d, ^3^*J*(H,H) = 7.0 Hz, 4H, Ar-H, *N*-benzoyl); ^13^C NMR (CD_3_OD): δ 46.01 (s, CH_2_), 51.34 (d, ^2^*J*(P,C) = 5.1 Hz, CH_2_), 117.92 (s), 121.46 (s), 128.90 (s), 129.17 (s), 129.66 (s), 130.08 (s), 130.46 (s), 130.77 (s), 133.74 (s), 133.90 (s), 134.93 (d, ^3^*J*(P,C) = 10.2 Hz, ipso-C), 152.99 (s), 171.37 (s, C=O); FT-IR (KBr): 3431 (NH), 3064 (CH), 2852 (CH), 2820 (CH), 1667 (C=O), 1596, 1454, 1377, 1326, 1190 (P=O), 1132 (P-N_amine_), 965(P-N_amide_), 759, 688, 572 (Sn-C)_as_, 532 (Sn-C)_s_ cm^−1^.

#### Bis[tris(4-phenylpiperazin-1-yl)phosphinic amide-κ*O*]dichlorido dimethyltin(IV) (**4**)

Tris(4-phenylpiperazin-1-yl)phosphinic amide (1 mmol, 0.530 g) was added to a solution of dimethyltin(IV) dichloride (0.5 mmol, 0.110 g) in dry methanol (15 mL) at room temperature, and the mixture was stirred for two hours. Then the solution flask was placed in an ultrasonic bath at 30 °C for one hour. The evaporation of the solvent gave the nanoparticles of the product, which were further purified by washing with diethylether and CCl_4_. mp 145–147 °C; yield: 61%; anal. Calcd for C_62_H_84_Cl_2_N_12_O_2_P_2_Sn: C, 58.13; H, 6.61; N, 13.12; found: C, 58.14; H, 6.60; N, 13.13; ^31^P{^1^H} NMR (DMSO-*d*_6_): δ 17.33 (s); ^1^H NMR (DMSO-*d*_6_): δ 0.97 (d, ^2^*J*(^119^Sn, H) = 83.4 Hz, Sn-CH_3_), 1.02 (s, 6H, Sn-CH_3_), 3.09 (m, 24H, ring-CH_2_), 3.19 (m, 24H, ring-CH_2_), 6.79 (t, ^3^*J*(H,H) = 8.0 Hz, 6H, Ar-H), 6.96 (d, ^3^*J*(H,H) = 8.0 Hz, 12H, Ar-H), 7.22 (t, ^3^*J*(H,H) = 8.0 Hz, 12H, Ar-H); ^13^C NMR (CD_3_OD): δ 22.94 (s, Sn-CH_3_), 45.71 (s, ring-CH_2_), 49.07 (d, ^2^*J*(P,C) = 5.7 Hz, ring-CH_2_), 115.73 (s), 119.20 (s), 128.91 (s), 151.21 (s), 165.57 (s, C=O); FT-IR (KBr): 2914 (CH), 2822 (CH), 1597, 1499, 1450, 1231, 1135 (P=O), 968 (P-N), 759, 635 (Sn-C)_as_, 529 (Sn-C)_s_ cm^−1^.

## Conclusion

Four new organotin(IV) complexes with phosphoric triamide ligands were synthesized and characterized by ^1^H, ^31^P, ^13^C NMR, IR, UV, fluorescence spectroscopy and elemental analysis. According to the ^1^H NMR spectra, it was concluded that the Sn atoms adopt octahedral conformations. The geminal Sn, H coupling constants ^2^*J*(^119^Sn,H) = 55.1, 83.4 Hz, were measured in the ^1^H NMR spectra of complexes **1** and **4**. Using ultrasonication, spherical nanoparticles of complexes **1**–**4** were prepared, and their SEM micrographs indicate that the nanoparticle sizes are about 20–25 nm. The fluorescence spectra illustrate blue shifts for the λ(max) of emission and a decrease in the peak intensities upon complexation.

## References

[1] Priscila Naidek K, Bianconi F, Rizuti da Rocha T C, Zanchet D, Alves Bonacin J, Novak M A, das Graças Fialho Vaz M, Winnischofer H (2011). J Colloid Interface Sci.

[2] Li Z-W, Li X-H, Tao X-J, Zhang Z-J, Yu L-G (2012). Mater Lett.

[3] Zhu L, Zheng X, Liu X, Zhang X, Xie Y (2004). J Colloid Interface Sci.

[4] Cassani M C, Davis M J, Hitchcock P B, Lappert M F (2005). Inorg Chim Acta.

[5] Denmark S E, Fu J (2003). J Am Chem Soc.

[6] Zhang R, Sun J, Ma C (2004). Inorg Chim Acta.

[7] Zhu C, Yang L, Li D, Zhang Q, Dou J, Wang D (2011). Inorg Chim Acta.

[8] Ruan B, Tian Y, Zhou H, Wu J, Hu R, Zhu C, Yang J, Zhu H (2011). Inorg Chim Acta.

[9] Xanthopoulou M N, Hadjikakou S K, Hadjiliadis N, Schürmann M, Jurkschat K, Michaelides A, Skoulika S, Bakas T, Binolis J, Karkabounas S (2003). J Inorg Biochem.

[10] Matsuno-Yagi A, Hatefi Y (1993). J Biol Chem.

[11] Denmark S E, Su X (1999). Tetrahedron.

[12] Munguia T, López-Cardoso M, Cervantes-Lee F, Pannell K H (2007). Inorg Chem.

[13] Yoder C H, Margolis L A, Horne J M (2001). J Organomet Chem.

[14] Murugavel R, Pothiraja R, Shanmugan S, Singh N, Butcher R J (2007). J Organomet Chem.

[15] Pellerito L, Nagy L (2002). Coord Chem Rev.

[16] Sanhoury M A, Ben Dhia M T, Khaddar M R (2011). J Fluorine Chem.

[17] Gholivand K, Shariatinia Z, Pourayoubi M (2006). Polyhedron.

[18] Gholivand K, Shariatinia Z (2006). J Organomet Chem.

[19] Ben Dhia M T, Sanhoury M A K, Essalah K, Khaddar M R (2011). Phosphorus, Sulfur Silicon Relat Elem.

[20] Gholivand K, Oroujzadeh N, Afshar F (2010). J Organomet Chem.

[21] Gholivand K, Farshadian S, Hosseini Z, Khajeh K, Akbari N (2010). Appl Organomet Chem.

[22] Gholivand K, Farshadian S, Hosseini Z (2012). J Organomet Chem.

[23] Silvestru C, Rösler R, Silvestru A, Drake J E (2002). J Organomet Chem.

[24] Sevcik R, Necas M, Novasad J (2003). Polyhedron.

[25] Varga R A, Schuerman M, Silvestru C (2001). J Organomet Chem.

[26] Safarifard V, Morsali A (2012). Ultrason Sonochem.

[27] Aboutorabi L, Morsali A (2011). Ultrason Sonochem.

[28] Sadeghzadeh H, Morsali A (2011). Ultrason Sonochem.

[29] Soltanzadeh N, Morsali A (2010). Ultrason Sonochem.

[30] Ramazani M, Morsali A (2011). Ultrason Sonochem.

[31] Shariatinia Z, Asadi E, Yousefi M, Sohrabi M (2012). J Organomet Chem.

[32] Shariatinia Z, Védova C O D, Erben M F, Tavasolinasab V, Gholivand K (2012). J Mol Struct.

[33] Gielen M, Jurkschat K, Meunier-Piret J, van Meerssche M (1984). Bull Soc Chim Belg.

[34] Altmann R, Jurkschat K, Schürmann M, Dakternieks D, Duthie A (1998). Organometallics.

[35] Jurkschat K, Hesselbarth F, Dargatz M, Lehmann J, Kleinpeter E, Tzschach A, Meunier-Piret J (1990). J Organomet Chem.

